# Carbon Monoxide Poisoning: Diagnosis, Prognostic Factors, Treatment Strategies, and Future Perspectives

**DOI:** 10.3390/diagnostics15050581

**Published:** 2025-02-27

**Authors:** Mohd Afzal, Shagun Agarwal, Rabab H. Elshaikh, Asaad M. A. Babker, Ranjay Kumar Choudhary, Pranav Kumar Prabhakar, Farhana Zahir, Ashok Kumar Sah

**Affiliations:** 1 Department of Medical Laboratory Technology, Arogyam Institute of Paramedical & Allied Sciences (Affiliated to H.N.B.Uttarakhand Medical Education University), Roorkee 247661, India; afzalglocal@gmail.com; 2 School of Allied Health Sciences, Galgotias University, Greater Noida 203201, India; shagunmpt@gmail.com; 3 Department of Medical Laboratory Sciences, College of Applied & Health Sciences, A’ Sharqiyah University, Ibra 400, Oman; rabab.mahmoud@asu.edu.om; 4 Department of Medical Laboratory Sciences, College of Health Sciences, Gulf Medical University, Ajman 4184, United Arab Emirates; azad.88@hotmail.com; 5 Department of Medical Laboratory Technology, Amity Medical School, Amity University Haryana, Gurugram 122412, India; r.choudharymt@gmail.com; 6 Parul Institute of Applied Sciences & Research and Development Cell, Parul University, Vadodara 391760, India; prabhakar.iitm@gmail.com; 7 Department of Biology, College of Science, Qassim University, Buraidah 51452, Saudi Arabia; farhanazahir@gmail.com

**Keywords:** carbon monoxide poisoning, biomarkers, hyperbaric oxygenation, public health

## Abstract

Carbon monoxide (CO) poisoning is a significant public health issue, with diagnosis often complicated by non-specific symptoms and limited access to specialised tools. Early detection is vital for preventing long-term complications. The review examines diagnostic challenges, prognostic factors, management strategies, and future advancements in CO poisoning. It highlights the limitations of current diagnostic techniques such as blood carboxyhaemoglobin levels and pulse CO-oximetry, while exploring emerging methods for rapid detection. Prognosis is influenced by exposure severity and delayed treatment, which increases the risk of neurological damage. Hyperbaric oxygen therapy (HBOT) remains the primary treatment but is not always accessible. Advances in portable CO-oximeters and biomarkers offer potential for improved early diagnosis and monitoring. Addressing resource limitations and refining treatment protocols are crucial for better patient outcomes. Future research should focus on personalised management strategies and the integration of modern technologies to enhance care.

## 1. Introduction

Carbon monoxide (CO) poisoning is a critical public health concern due to its high morbidity and mortality rates [1]. CO is a colourless, odourless, and tasteless gas, making it extremely insidious as individuals may be unaware of exposure until symptoms emerge [2]. Once breathed, CO bonds with haemoglobin to produce carboxyhaemoglobin, limiting oxygen delivery and leading to tissue hypoxia. This can result in serious health implications, including brain impairment and death.

CO poisoning is a major issue across the world [3]. According to previous research by Mattiuzzi C. et al., the global cumulative incidence and mortality rates for carbon monoxide (CO) poisoning are currently estimated at 137 cases and 4.6 deaths per million people, respectively. Over the past 25 years, the incidence has remained stable, while mortality rates and the proportion of patients who die have decreased by 36% and 40%, respectively. The incidence of CO poisoning is consistent across sexes, although mortality is twice as high in men. Incidence rates show two notable peaks: one in children aged 0–14 years and another in adults aged 20–39 years. Mortality rates consistently increase with age, peaking in individuals aged 80 years or older. The incidence of CO poisoning rises in parallel with the sociodemographic index (SDI), although further analysis is needed to validate these findings. Mortality follows a similar pattern, being approximately 2.1 and 3.6 times higher in middle- and middle-to-high-SDI countries compared to low-to-middle-SDI countries. In conclusion, while global data suggest that the burden of CO poisoning remains stable, with a consistent decline in both fatal outcomes and mortality rates over the past 25 years, the reliability of primary data sources in many countries, particularly with respect to accurate diagnosis of CO poisoning, remains uncertain. Therefore, caution is needed, and further field studies, especially in lower-income countries, are essential [1,4].

In the United States, CO poisoning affects around 50,000 people each year, with a fatality incidence ranging from 1% to 3% [5]. In 2022, tentative statistics from the Centers for Disease Control and Prevention (CDC) showed 1244 fatalities due to CO poisoning, underlining its continued concern [6]. CO poisoning can cause a variety of symptoms, including headaches, dizziness, weakness, nausea, vomiting, chest pain, and confusion. Severe exposure can result in loss of consciousness, arrhythmias, seizures, and death. Because these symptoms are non-specific, it is critical to be aware of them and recognise them as soon as possible to avoid negative outcomes [7].

### Sources and Incidence of Exposure

CO is created by the incomplete combustion of carbon-containing fuels such as petrol, natural gas, oil, coal, and wood [8].

Common sources of CO exposure are: Residential heating systems: Malfunctioning or inadequately vented furnaces and heaters can emit CO into residential rooms [9].

Automobile exhaust: Running vehicles in enclosed spaces, such as garages, can lead to dangerous CO accumulation [10].

Portable generators and engines: Use of generators or gasoline-powered tools indoors or near open windows can result in CO buildup [11].

Cooking appliances: Charcoal grills and gas stoves used indoors without adequate ventilation pose significant risks [12].

The prevalence of CO poisoning varies by area and is influenced by climate, socioeconomic level, and public perception. In the United States, non-fire-related CO exposures account for the vast majority of instances. Certain groups, such as the elderly, children, and those with pre-existing medical issues, are especially vulnerable to CO poisoning. Lower-income households may rely on alternate heating methods, which increases the risk of CO exposure [13,14]. Seasonal differences can influence incidence rates. During the winter months, people use more heating appliances, which raises their risk of CO exposure. For example, a paper emphasised the hazards of utilising charcoal grills indoors for warmth, which can lead to CO poisoning. Similarly, the usage of gas heaters and stoves without adequate ventilation has been connected to CO poisoning cases [5].

This study aims to give a complete examination of carbon monoxide (CO) poisoning, with an emphasis on diagnosis, prognosis, and treatment. This review attempts to improve understanding of CO poisoning’s pathogenesis and devastating impact on human health by synthesising the most recent studies and therapeutic guidelines.

## 2. Pathophysiology of Carbon Monoxide Poisoning

### 2.1. Mechanism of Toxicity: Interaction with Haemoglobin and Cellular Effects

Carbon monoxide (CO) is hazardous chiefly due to its interaction with haemoglobin and disruption of cellular functions. CO binds to haemoglobin with around 200–250 times the affinity of oxygen, resulting in carboxyhaemoglobin (COHb). This binding lowers the blood’s oxygen-carrying capacity and decreases oxygen delivery to tissues. Furthermore, COHb modifies the conformation of the haemoglobin molecule, enhancing its affinity for oxygen at the remaining binding sites. This change in the oxygen dissociation curve impedes oxygen delivery to peripheral tissues, exacerbating the symptoms of hypoxia [5,15].

Beyond its effect on haemoglobin, CO directly inhibits cellular respiration by attaching to cytochrome c oxidase in the mitochondrial electron transport chain. This inhibits oxidative phosphorylation, resulting in decreased adenosine triphosphate (ATP) synthesis and an increased reliance on anaerobic metabolism. Lactic acidosis exacerbates cellular and systemic dysfunction [16].

CO poisoning causes oxidative stress by releasing reactive oxygen species (ROS) and activating inflammatory pathways. These effects can induce lipid peroxidation, protein degradation, and apoptosis, especially in highly oxygen-demanding organs like the brain and heart. The combination hypoxia and oxidative damage causes the neurological and cardiovascular symptoms that are hallmarks of CO poisoning [5,17].

### 2.2. Mechanisms of Cardiac Injury in Carbon Monoxide Poisoning

Carbon monoxide (CO) poisoning is closely linked to a wide range of cardiac complications, including acute myocardial damage, severe necrosis, and contractile dysfunction. This link was established when Klebs initially documented heart rate variations in CO intoxication in 1865 [18,19]. Myocardial tissue, which is extremely vulnerable to oxygen deprivation, experiences hypoxic damage that is aggravated by increased oxygen demand due to increased contractility, decreased coronary reserve, and hindered cellular respiration [20]. Beyond these effects, CO causes cellular and subcellular damage to the heart. Fracasso et al. recently found indications of localised heart injury in the right ventricle, as shown by increased fibronectin and the terminal complement complex C5b9 [21].

CO binds to the heme group in myoglobin with 60 times more affinity than oxygen, limiting oxygen delivery to mitochondria and oxidative phosphorylation [5]. This disturbance compels cardiomyocytes to use anaerobic metabolism, resulting in hypoxia, lactic acidosis, and death [22]. CO also impairs mitochondrial activity by interfering with cytochrome c oxidase and lowering glutathione (GSH) levels, limiting ATP generation [23]. Elevated CO levels activate apoptotic enzymes like caspase-1, which causes endothelial cell death and promotes nitrosative stress, lipid peroxidation, and inflammation [24]. Additionally, CO has been demonstrated to cause transcapillary efflux, leukocyte sequestration, and oxidation of plasma low-density lipoproteins, which increases endothelial damage and coronary vasoconstriction [20].

### 2.3. Functional and Structural Cardiac Consequences

CO poisoning has considerable prothrombotic effects, as evidenced by multiple research and case reports linking it to arterial and venous thrombosis, including stent thrombosis [25]. It also causes anatomical alterations such as myocardial fibrosis and poor left ventricular function [26]. Prolonged CO exposure can cause functional impairments, including reduced sarcoplasmic reticulum Ca^2+^ ATPase (SERCA-2a) expression and disrupted Ca^2+^ reuptake [27]. This leads to increased intracellular Ca^2+^ levels, heightened myofilament sensitivity, and hyperadrenergic states, which increase the risk of arrhythmia [28].

Ischaemic myocardial injury is exacerbated by peripheral circulatory failure and hypotension, which both limit left ventricular output and tissue oxygenation [20]. Severe cardiac decompensation caused by CO exposure can eventually lead to multiorgan failure and is a leading cause of death in seriously poisoned people. Furthermore, extended CO exposure has been associated with increased oxidative stress and decreased coronary perfusion, demonstrating its wide and serious influence on cardiovascular health [29].

### 2.4. Pathophysiology of Brain Injuries in Acute Carbon Monoxide Poisoning

The clinical manifestations of CO poisoning range from mild symptoms like headache, dizziness, and nausea to severe effects such as confusion and delayed neuropsychiatric issues. They underscore the importance of early recognition and intervention to prevent long-term complications.

Acute carbon monoxide (CO) poisoning has a profound impact on the central nervous system (CNS), with symptoms ranging in severity according to the intensity and duration of CO exposure. During the acute phase, patients may have headaches, dizziness, syncope, seizures, brain infarction, and loss of consciousness [30]. Following recovery, some individuals suffer delayed neurological sequelae (DNS), including cognitive impairments, Parkinsonism, motor abnormalities, and peripheral neuropathy [31].

MRI and CT scans were used to identify common brain lesions in the basal ganglia, hippocampus, and corpus callosum during the acute phase [32]. DNS, on the other hand, has been linked to profound white matter damage. Acute brain injuries are linked to elevated levels of biochemical markers such as neuron-specific enolase (NSE) and S100β, as well as increased cytokines, interleukins, and growth factors in patients experiencing loss of consciousness [30,33].

CO poisoning affects the dopaminergic system, causing an increase in extracellular dopamine levels by increasing release while inhibiting metabolism and reuptake. This dopamine excess lasts for weeks and is linked to oxidative damage, apoptosis, and the death of synapses and nuclei in the mesolimbic system, notably in the globus pallidus [34]. Researchers have also connected dopamine’s oxidative byproducts, like reactive quinones and oxygen species, to DNS formation.

Dopamine overproduction causes striatal lesions that mirror neurotoxicity found in methamphetamine or MDMA misuse situations [35,36]. Additionally, toxic leukoencephalopathy, characterised by cerebral demyelination and structural abnormalities in deep white matter, has been described in CO poisoning patients, however the particular cellular targets of CO are unknown [37].

## 3. Clinical Manifestations of Carbon Monoxide Poisoning

The symptoms of carbon monoxide (CO) poisoning vary greatly depending on exposure length and CO levels and are frequently vague, making early detection crucial for effective diagnosis and treatment. Symptoms might range from minor flu-like symptoms to serious neurological and cardiovascular consequences, coma, or death. Because of their high metabolic needs, the brain and heart are especially susceptible to CO poisoning [38].

### 3.1. Neurological Effects

The most noticeable clinical signs of CO poisoning are neurological problems [32]. A headache, often characterised as frontal and either dull or throbbing, is the most prevalent presenting symptom, accounting for up to 84% of cases [39,40]. However, the intensity of the headache is not related to carboxyhaemoglobin (COHb) levels. Dizziness is common with headaches and has been found in up to 92% of people exposed to CO [41]. As CO exposure rises, more severe neurological symptoms may emerge, such as disorientation, fainting, seizures, stroke-like syndromes, and coma [42].

Memory loss, attention difficulties, poor executive function, slower mental processing, and anxiety or depression are all cognitive and neurobehavioural sequelae that might last a year or more in some individuals [43]. Long-term effects are associated with loss of consciousness during the acute phase, age above 36, and COHb levels more than 25% [44,45]. Delayed neurological sequelae (DNS), which can develop days or months after the original poisoning, can cause symptoms ranging from minor cognitive impairments to severe dementia, hallucinations, movement abnormalities, or incontinence [46].

While DNS resolve in roughly 75% of cases without special treatment, hyperbaric oxygen (HBO) therapy has been used with various degrees of efficacy to prevent or manage them. Brain imaging frequently indicates abnormalities in the basal ganglia (particularly in the globus pallidus) as well as corpus callosum atrophy in severe instances [47].

### 3.2. Cardiovascular and Other Systemic Effects

Cardiovascular problems are typical with CO poisoning. Tachycardia, a compensatory reaction to systemic hypoxia, is commonly seen. Even low COHb levels can exacerbate myocardial ischaemia, and cardiac necrosis can develop in the absence of obvious symptoms [48]. Acute exposure can cause high cardiac biomarkers, despite intact coronary arteries, as well as mild to severe left ventricular dysfunction, which is related to COHb levels and exposure time. Additionally, CO-induced alterations can affect cardiac conduction, resulting in arrhythmias, however, these do not always correspond with COHb levels [49].

Pregnant women and their foetuses are more vulnerable to CO poisoning because foetal tissues are more sensitive to hypoxia, and foetal haemoglobin binds CO more strongly than adult haemoglobin [50]. This variation can lead to more protracted CO elimination in the foetus, making even low-level exposure potentially hazardous. Prolonged oxygen treatment (HBO) is frequently necessary to reduce foetal hazards [51].

Other systemic effects of CO poisoning include rhabdomyolysis, renal failure, non-cardiogenic pulmonary oedema, and the formation of cutaneous blisters [52]. The typical “cherry-red” skin discolouration, while historically associated with CO poisoning, is rarely seen in clinical practice. Early detection and management, including oxygen treatment, are critical for reducing these varied and possibly life-threatening consequences [53].

## 4. Diagnosis of Carbon Monoxide Poisoning

A complete clinical examination, a full patient history, and the identification of probable exposure sources and risk factors are required for an accurate diagnosis [54].

### 4.1. Clinical Assessment and History

Patients with CO poisoning sometimes present with vague symptoms, making diagnosis difficult. Common symptoms include headaches, dizziness, weakness, nausea, disorientation, and changed mental state. Severe instances may cause loss of consciousness or death. Tachycardia, tachypnea, and hypotension may be detected during a physical examination. Given the diversity of symptoms, a high level of suspicion is required, especially when numerous people in the same area report identical concerns [54,55].

#### Identifying Risk Factors and Exposure Sources

Carbon monoxide is created when carbon-containing fuels are not burned completely. Common causes include faulty furnaces, gas stoves, water heaters, fireplaces, and automobiles. Indoor usage of charcoal barbecues or portable generators can potentially result in harmful CO levels [56].

Carbon monoxide (CO) poisoning risk factors include age, with infants and the elderly being more vulnerable; pre-existing health conditions such as chronic heart disease, anaemia, or respiratory issues, which increase susceptibility; and environmental factors such as poorly ventilated spaces, especially during the colder months when heating appliances are frequently used. Recognising these risk factors and possible exposure sources is critical for timely diagnosis and action [57].

To summarise, diagnosing CO poisoning necessitates a thorough clinical examination, a complete patient history, and an understanding of environmental risk factors and exposure sources. Early detection and treatment are critical to avoiding serious results [52].

### 4.2. Diagnostic Tools for Carbon Monoxide Poisoning

Carbon monoxide (CO) poisoning may be accurately diagnosed using appropriate diagnostic equipment. Pulse CO-oximetry is a rapid, non-invasive method of measuring carboxyhaemoglobin (COHb) levels, however, it may be less precise than arterial blood samples. The gold standard for measuring COHb levels is arterial blood gas (ABG) measurement, which validates the severity of the poisoning. Traditional pulse oximetry is unreliable because it cannot distinguish COHb from oxyhaemoglobin. In extreme situations, imaging such as CT or MRI can assist assess cerebral damage caused by hypoxia, but it cannot directly diagnose CO poisoning. Early application of these technologies is critical for successful therapy [38,52].

Carboxyhaemoglobin forms when carbon monoxide binds to haemoglobin, limiting oxygen delivery and causing hypoxic damage even at normal oxygen saturation levels. Even low COHb concentrations can be life-threatening, hence precise measurement is critical [15]. Pulse CO-oximeters (Masimo Corp, Irvin, CA, USA) employ a variety of light wavelengths to distinguish between oxyhaemoglobin, deoxyhaemoglobin, carboxyhaemoglobin, and methaemoglobin. Light travels through tissue (often a fingertip), and absorption at various wavelengths determines COHb concentrations. Although quick and non-invasive, accuracy may be impaired in cases of poor perfusion or motion artefacts [58,59].

ABG with CO-oximetry is an excellent method for determining COHb concentrations. Unlike normal ABG, which measures partial pressures of gases, CO-oximetry employs spectrophotometric analysis to directly quantify the proportions of various haemoglobin species [60].

Detection is challenging as standard pulse oximeters cannot distinguish between oxyhaemoglobin and carboxyhaemoglobin, resulting in falsely normal or high oxygen saturation levels. Because haemoglobin has a high affinity for carbon monoxide, even low exposure can severely impede oxygen transport, emphasising the importance of precision testing procedures [52].

#### 4.2.1. Carboxyhaemoglobin Levels

Carboxyhaemoglobin (COHb) is created when carbon monoxide (CO) binds to haemoglobin and prevents oxygen delivery in the blood. COHb levels are crucial in detecting carbon monoxide poisoning, with typical values of less than 2% in non-smokers and 5–10% in smokers [61]. Levels above 10–15% suggest poisoning, while levels above 25% are serious and potentially fatal. COHb values can help determine poisoning severity and guide treatment methods [15].

#### 4.2.2. Pulse CO-Oximetry

Pulse CO-oximetry is a non-invasive diagnostic method that calculates COHb levels by measuring light absorption at various wavelengths [58].

***Advantages:*** Pulse CO-oximetry has several advantages, including its speed and ease of use in emergency or mass casualty scenarios, its value for continuous monitoring of COHb levels, and its non-invasive nature, which makes it less uncomfortable and convenient for patients [59].

***Disadvantages:*** Pulse CO-oximetry has the drawback of being less accurate than arterial blood gas (ABG) analysis, as well as being susceptible to erroneous results produced by motion artefacts, nail polish, or ambient light [62].

#### 4.2.3. Arterial Blood Gas Analysis

ABG analysis is the gold standard for determining COHb concentrations. It entails collecting an arterial blood sample and analysing it using a CO-oximeter [60].

***Advantages:*** The benefits of arterial blood gas (ABG) analysis include the capacity to offer precise and direct measurements of COHb levels as well as the ability to examine other vital factors such as pH, oxygen, and carbon dioxide levels, all of which are critical in severe instances [63].

***Disadvantages:*** The downsides of arterial blood gas (ABG) analysis include the fact that it is invasive, requires skilled staff to conduct, and takes longer than pulse CO-oximetry [64].

### 4.3. Comparison and Modern Perspective

Pulse CO-oximetry is useful for fast screening, especially in pre-hospital situations, however, ABG analysis is still required for verifying diagnosis and treating severe cases. Modern improvements aim to improve the precision and mobility of non-invasive technologies, hence increasing their dependability in clinical and field applications [65].

### 4.4. Challenges in Early Detection of Carbon Monoxide (CO) Poisoning

***Non-specific symptoms:*** Early symptoms of CO poisoning (headache, dizziness, nausea, and disorientation) are sometimes ambiguous and can be confused for other common ailments including the flu, viral infections, or food poisoning. This makes it difficult to detect CO poisoning, especially in its early stages [66].

***Normal oxygen saturation levels:*** Traditional pulse oximetry measures oxygen saturation but does not discriminate between oxyhaemoglobin and carboxyhaemoglobin (COHb). As a result, CO poisoning may go undetected in individuals with normal oxygen saturation levels, even if their COHb values are excessive, resulting in a delayed diagnosis [67].

***Lack of awareness in low-level exposure***: Chronic or low-level CO exposure can induce modest symptoms or be asymptomatic, sometimes going untreated until more serious consequences arise, such as long-term brain damage. People who live in poorly ventilated places or have specific health issues may be more vulnerable since they are unaware of the dangers of CO exposure [68].

***Inadequate diagnostic tools in field settings:*** In emergency or rural settings, sophisticated diagnostic technologies like ABG or pulse CO-oximetry may be unavailable. Without access to specialised equipment for testing COHb levels, the initial diagnosis may be delayed, resulting in inefficient therapy and poor results [62].

***Variation in individual susceptibility:*** Individuals may suffer variable levels of symptoms at comparable COHb values, depending on factors such as age, underlying health issues (e.g., heart disease or anaemia), and general tolerance to hypoxia. This heterogeneity affects early detection and diagnostic procedures [69].

***Environmental and occupational exposure:*** When symptoms are moderate or sporadic, chronic or recurrent exposure to CO (from sources like heating systems, generators, or vehicle exhaust) may go unnoticed as a possible cause of poisoning in industrial, residential, or recreational contexts. This may create a delay in determining the actual source of health problems, particularly for employees exposed to CO in poorly ventilated settings [70].

Limited public health screening: Routine screening for CO exposure is not included in health evaluations, which results in lost chances for early identification, particularly in high-risk populations (the elderly, babies, and those with pre-existing respiratory or cardiac disorders) [71].

***False negative results:*** Diagnostic methods such as pulse CO-oximetry or exhaled CO measurement may provide false negatives in situations of extremely low COHb levels or in people with chronic CO exposure who have acclimated to reduced oxygen levels. This might result in an underestimate of CO poisoning severity [72].

Addressing the challenges of carbon monoxide (CO) poisoning necessitates many critical solutions. First, education and awareness are critical for increasing public and healthcare professionals’ understanding of CO poisoning symptoms, hazards, and preventative actions, resulting in early detection [73]. Enhanced diagnostics play an important role in increasing early detection by creating more sensitive, portable, and accessible diagnostic equipment, such as wearable COHb sensors. Environmental monitoring using CO detectors in high-risk areas such as homes, businesses, and public spaces can offer real-time notifications of possible exposure. Finally, frequent screening in high-risk populations, such as the elderly, children, and those with heart or respiratory issues, can aid in the early detection of CO poisoning, particularly in situations of low-level exposure [74].

## 5. Prognosis: Factors Affecting Outcomes in Carbon Monoxide (CO) Poisoning

### 5.1. Severity of Exposure

The severity of CO poisoning is determined by the quantity of carbon monoxide in the atmosphere and the time of exposure [75]. Higher CO concentrations and longer exposure durations cause more severe poisoning, raising the possibility of long-term brain impairment, organ failure, or death. For example, exposure to CO concentrations of more than 30% can cause loss of consciousness, and concentrations greater than 50% can be lethal if not treated immediately [76].

### 5.2. Promptness of Treatment

The speed with which therapy is delivered has a substantial impact on CO poisoning outcomes [77]. Early detection and management, particularly with 100% oxygen or hyperbaric oxygen treatment (HBOT), can aid in the removal of CO from haemoglobin and tissues, lowering the risk of long-term harm [78]. Delays in treatment increase the likelihood of irreparable damage, particularly to the brain and heart, and can worsen the prognosis [79].

### 5.3. Pre-Existing Health Conditions

Individuals with underlying health concerns, such as cardiovascular illness, respiratory diseases (e.g., COPD, asthma), anaemia, or neurological abnormalities, are more likely to experience catastrophic results when exposed to CO [80]. These illnesses can increase the consequences of CO poisoning by impairing the body’s capacity to deal with low oxygen levels [81]. For example, people with heart disease may face more severe cardiovascular consequences as a result of the additional stress of CO exposure, whereas those with anaemia may have a lower capacity to transfer oxygen even under normal settings [82].

### 5.4. Long-Term Effects and Recovery from Carbon Monoxide (CO) Poisoning

Neurological effects: One of the most serious long-term consequences of CO poisoning is brain impairment. Prolonged exposure to CO can cause cognitive impairment, memory loss, personality changes, and difficulties focussing [83]. Even after the initial phase is over, some people may continue to have headaches, dizziness, and other cognitive problems. Severe instances might result in lasting brain damage, such as encephalopathy or other mental illnesses [84].

***Cardiovascular effects:*** CO poisoning can have long-lasting effects on the cardiovascular system. In extreme cases of poisoning, the heart muscle might be damaged, increasing the risk of arrhythmias or myocardial infarctions [85]. This is especially true for people who already have cardiac issues, since they may be more susceptible to these consequences. Even after recovering from acute poisoning, individuals may develop long-term cardiac problems [86].

***Respiratory issues:*** While CO predominantly affects the cardiovascular and neurological systems, it can also cause chronic respiratory difficulties [87]. Chronic exposure, even at low levels, can exacerbate illnesses such as asthma and chronic obstructive lung disease (COPD) [83]. Individuals who have been exposed to severe CO poisoning may develop impaired lung function and continuing shortness of breath [88].

***Recovery process:*** Recovery from CO poisoning varies greatly depending on the degree of the poisoning, the timing of therapy, and the individual’s general health [89]. Most people will recover completely from mild to moderate poisoning after receiving adequate treatment, such as oxygen therapy. Individuals with severe poisoning or delayed treatment may require a prolonged recovery period and therapy for cognitive or physical impairments [90]. Long-term impacts may be addressed through rehabilitation programs that include physical treatment, cognitive therapy, and psychological counselling [91].

**Psychological effects:** CO poisoning often results in long-term psychological symptoms such as sadness, anxiety, and post-traumatic stress disorder (PTSD) [92]. These psychological impacts might result from the stress of the poisoning, persistent health difficulties, or abnormalities in cognitive and neurological function. Mental health support and treatment are critical parts of the rehabilitation process [93].

***Impact on quality of life:*** Even when treated, CO poisoning can have a long-term impact on a person’s quality of life. Patients may have long-term weariness, difficulties with everyday duties, and a general decline in their capacity to perform routine living activities [94]. Continuous assistance from healthcare practitioners, family, and support networks is frequently required to help patients adjust to any changes in their health [95].

## 6. Management Strategies for Carbon Monoxide (CO) Poisoning

**Figure 1:**
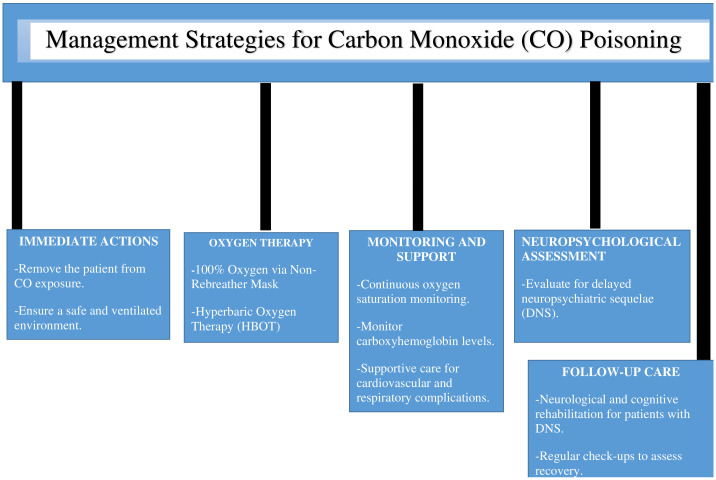
Management Strategies for Carbon Monoxide (CO) Poisoning.

Figure 1 highlights the importance of monitoring carboxyhaemoglobin levels, managing complications, and assessing for delayed neuropsychiatric sequelae. Preventive measures, such as CO detectors and public awareness, and follow-up care for cognitive and neurological recovery are also included.

### 6.1. Immediate Interventions

Immediate intervention is required in the treatment of CO poisoning to reduce the consequences of hypoxia and avoid organ damage [96]. As soon as CO poisoning is detected, patients should be evacuated from the source of exposure to prevent further CO intake. While transferring the patient to a medical institution, emergency treatment to preserve vital signs is critical, including sustaining respiratory and cardiovascular function as needed [97]. Individuals who are unconscious or in respiratory distress may require sophisticated airway treatment (e.g., intubation) [98].

### 6.2. Removal from Source of Exposure

The first step in treating CO poisoning is to remove the patient from the polluted surroundings. This entails removing the individual from the source of exposure (such as a poorly ventilated room, an automobile, or an industrial environment) and quickly transporting them to fresh air [99]. In a medical environment, this might entail relocating the patient to a designated place with appropriate ventilation to avoid further CO inhalation [100]. The sooner an individual is removed from the exposure source, the lower the danger of severe or long-term consequences [101].

### 6.3. Oxygen Therapy (100% Oxygen)

The primary therapy for CO poisoning is to administer 100% oxygen. High-concentration oxygen helps to rapidly displace CO from haemoglobin, enabling the return of normal oxygen-carrying capacity in the blood [102]. Oxygen therapy also speeds up the removal of CO from the body, as CO is excreted through the lungs, with 70% of COHb cleared within the first hour of treatment [103]. In most circumstances, oxygen treatment is provided by a non-rebreather mask, but in extreme cases, an endotracheal tube is required [104]. Hyperbaric oxygen treatment (HBOT) may be advised for patients with severe poisoning, such as those who have lost consciousness or are experiencing critical symptoms. HBOT consists of supplying oxygen at higher-than-normal pressures, which greatly speeds the clearance of CO from the circulation and tissues and can lower the risk of long-term neurological damage [105]. HBOT, which includes inhaling pure oxygen in a pressurised chamber, is often used to treat decompression sickness, carbon monoxide poisoning, and chronic wounds. Many HBOT centres are located near water due to their origins in treating diving disorders, making coastal placement ideal for marine situations [106]. Coastal cities also have more hospitals and specialised services which promote hyperbaric medicine. Furthermore, the relaxing seaside atmosphere may assist in rehabilitation, however, clinical effectiveness remains paramount. While seaside HBOT centres made historical sense, today’s medical demands necessitate a more strategic distribution to improve healthcare accessibility and results [107].

### 6.4. Role of Hyperbaric Oxygen Therapy (HBOT)

Hyperbaric oxygen therapy (HBOT) is a primary therapeutic option for carbon monoxide (CO) poisoning. It entails administering 100% oxygen to the patient in a pressurised chamber, which greatly increases oxygen concentration in the circulation and improves tissue oxygenation [108]. The fundamental goal of HBOT is to remove carbon monoxide from haemoglobin and restore cellular oxygen supply, reducing the hypoxic consequences of CO poisoning [106].

***Indications for HBOT in CO poisoning***: HBOT is highly useful for moderate-to-severe carbon monoxide poisoning, particularly in patients who have significant neurological symptoms such as altered mental state, disorientation, coma, seizures, or chronic impairments [109]. It is also recommended for patients with high carboxyhaemoglobin levels (typically >25–30%), pregnant women due to the increased risk to foetal haemoglobin, people with cardiac complications such as myocardial ischaemia or arrhythmias, and cases of prolonged exposure or delayed symptoms, including delayed neurological sequelae (DNS), even after initial normobaric oxygen therapy [110].

### 6.5. Other Comprehensive Management Approaches for CO Poisoning

Supportive care: Supportive care is essential in the treatment of carbon monoxide (CO) poisoning, treating urgent requirements, and preventing complications [107].

***Oxygen therapy:*** To lower carboxyhaemoglobin levels and enhance oxygen delivery, the first-line therapy is to provide 100% oxygen using a non-rebreather mask or endotracheal intubation (if required) [111].

***Monitoring and stabilisation:*** Vital signs, oxygen saturation, and heart rhythms must be monitored on a continuous basis. Intravenous fluids may be administered to ensure haemodynamic stability [112].

***Seizure management:*** Anticonvulsants such as benzodiazepines are used to treat seizures caused by severe hypoxia [113].

***Temperature regulation:*** Hypothermia or hyperthermia should be treated immediately to avoid aggravating neurological or metabolic impairment [114].

**Nutritional and metabolic support:** Addressing metabolic acidosis with bicarbonate treatment and maintaining proper diet aids in recovery [115].

### 6.6. Managing Neurological and Cardiovascular Complications

Neurological complications: Neurological problems include delayed neurological sequelae (DNS) and cerebral oedema which are serious concerns after poisoning. DNS, which are characterised by cognitive deficits, personality changes, and motor dysfunction, can appear days or weeks after exposure [116]. Hyperbaric oxygen treatment (HBOT) is effective in preventing and controlling DNS, but long-term recovery frequently needs supporting therapies such as physical rehabilitation and cognitive therapy [117]. Mannitol or hypertonic saline can be used to successfully to treat high intracranial pressure in severe cerebral oedema situations [118].

**Cardiovascular complications:** Myocardial ischaemia and arrhythmias necessitate prompt hypoxia treatment by oxygen therapy to minimise cardiac stress, with antiarrhythmic medications delivered as needed for rhythm stabilisation [119]. Intravenous fluids and vasopressors such as norepinephrine are used to treat hypotension and shock, ensuring steady perfusion and blood pressure. Long-term cardiac surveillance is required for individuals with pre-existing cardiac problems or severe carbon monoxide exposure to avoid late-onset consequences like cardiomyopathy [120].

## 7. Laboratory Diagnostic Challenges

### 7.1. Limitations of Current Techniques

Table 1 provides a detailed comparison of various diagnostic tests used to detect carbon monoxide (CO) poisoning. The tests are evaluated based on their underlying principle, the time required for development or obtaining results, advantages such as ease of use or reliability, their sensitivity in detecting CO exposure, and potential limitations or drawbacks.

**Carboxyhaemoglobin (COHb):** Carboxyhaemoglobin (COHb) testing using blood gas analysers or spectrophotometry remains the primary diagnostic method for carbon monoxide (CO) poisoning. However, it has several severe drawbacks. COHb levels fall fast with oxygen treatment or termination of CO exposure, resulting in false negatives if testing is delayed [125]. Furthermore, COHb levels may not always correspond to the intensity of clinical symptoms, making reliable prediction of patient outcomes problematic [103]. Conventional pulse oximetry complicates the diagnosis since it cannot distinguish between oxyhaemoglobin and carboxyhaemoglobin, resulting in normal or deceptive oxygen saturation measurements in CO-poisoned individuals [126]. Furthermore, the absence of accurate and precise biomarkers to establish CO poisoning or determine the amount of tissue hypoxia complicates the diagnostic process [127].

### 7.2. Advances in COHb Testing and Diagnosis

Portable CO-oximeters, improved point-of-care devices that can measure COHb levels, improve early diagnosis and treatment of carbon monoxide poisoning [128]. Novel biomarkers, such as high lactate levels or oxidative stress and inflammatory indicators, have the potential to improve diagnostic and prognostic capacities [129]. Furthermore, the development of non-invasive technology, such as wearable sensors and breath analysis tools for real-time CO exposure detection, can overcome the limits of existing testing methods, boosting accessibility and prompt intervention [130].

### 7.3. Challenges in CO Poisoning Diagnosis in Resource-Limited Settings

Limited access to CO-oximetry and sophisticated diagnostic techniques in resource-constrained situations frequently leads to underdiagnosis and delayed treatment of carbon monoxide poisoning [123]. Cost limits make advanced diagnostic equipment expensive and unavailable in low-income communities, emphasising the need for simpler, more cost-effective alternatives. Furthermore, a shortage of skilled professionals in endemic areas impedes rapid and accurate diagnosis [131]. Inadequate healthcare infrastructure, particularly limited laboratory facilities and emergency services in rural and disadvantaged locations, exacerbates the ineffective handling of CO poisoning patients [132].

## 8. Prevention and Public Health Measures

***Installation of CO detectors:*** The installation of carbon monoxide (CO) detectors is a key step in preventing CO poisoning [133]. CO detectors are devices that monitor the air for the presence of carbon monoxide, an odourless, colourless, and tasteless gas, and issue early alerts before dangerous amounts develop [134]. Proper CO detector installation and maintenance considerably lower the risk of serious poisoning and death [135].

***Types of CO detectors:*** Battery-powered CO detectors are portable, simple to install, and excellent for fast deployment; nevertheless, they require regular battery replacement to function properly. Hardwired CO detectors are incorporated into the building’s electrical infrastructure and frequently have battery backups to ensure continuous functioning during power outages [136]. Plug-in CO detectors are intended to connect directly to electrical outlets, making them ideal for temporary or rental premises. They also have a battery backup for increased dependability. Smart CO detectors with Wi-Fi or Bluetooth connectivity offer sophisticated functionality by transmitting real-time warnings to smartphones or other connected devices, providing timely notifications even while the inhabitants are away [137].

Awareness gaps in low-resource situations frequently lead to families being ignorant of the necessity of CO detectors. Cost limits further limit their affordability in underserved regions, and maintenance negligence, such as failing to replace batteries or expired devices, jeopardises the usefulness of this life-saving equipment [138].

Governments and health organisations should encourage CO detector installation through public education campaigns and financial incentives. Enforcing regulations requiring CO detectors in residential and commercial buildings can increase adoption. Furthermore, programs that provide subsidised or free CO monitors in underprivileged populations can help to reduce CO-related health hazards [139].

### 8.1. Maintenance of Combustion Appliances

Regular maintenance of combustion appliances is an important preventive step against carbon monoxide poisoning. Combustion equipment, such as gas stoves, water heaters, furnaces, and fireplaces, can emit CO if not working correctly. Keeping these devices well-maintained can considerably lower the danger of CO accumulation in confined areas [140].

**Maintenance practices:** Annual inspections: Have a competent expert examine combustion appliances once a year to ensure they are running effectively and safely. This involves inspecting for any leaks, blockages, or evidence of wear that might result in incomplete combustion and CO emissions [141].

**Proper ventilation:** Ensure that appliances are properly ventilated to allow for the safe expulsion of CO and other combustion byproducts. Vents should be unobstructed and debris-free to keep CO from backing up into living rooms [142].

**Cleaning and servicing:** Regular cleaning of appliances, such as furnace filters and chimneys, may help avoid the accumulation of soot and debris, which can impede airflow and lead to unsafe CO concentrations [143].

**Carbon monoxide detectors**: Install CO detectors near combustion appliances to monitor CO levels and offer early alerts in case of a problem [144].

***Replacement of faulty components:*** If any elements of an appliance, such as gas valves, heat exchangers, or pilot lights, are broken or malfunctioning, they should be changed right away to avoid CO leakage [145].

***Importance of maintenance:*** Proper maintenance of combustion appliances reduces CO buildup by reducing the risk of leaks into indoor spaces, improves appliance efficiency by improving performance and lowering energy costs, and promotes safety by identifying potential hazards early enough to avoid life-threatening situations [146].

### 8.2. Public Awareness Campaigns

Public awareness campaigns are crucial in reducing carbon monoxide (CO) poisoning by informing communities about the hazards, preventative tactics, and safety precautions. Effective campaigns can drastically reduce CO-related events by using focused messages and outreach [147].

#### Strategies for Public Awareness Campaigns

***Educational programs:*** Conduct workshops, seminars, and school-based programs to educate the public on CO sources, signs of poisoning, and the necessity of preventive measures like CO detectors [148].

***Media outreach:*** Use television, radio, newspapers, and internet media to raise awareness about CO hazards and safety precautions, reaching a larger audience [149].

***Community engagement:*** Collaborate with local health organisations, community leaders, and non-governmental organisations (NGOs) to organise awareness campaigns, distribute instructional booklets, and install CO detectors in susceptible locations [150].

***Highlight real-life cases:*** Sharing real-life cases of CO poisoning can highlight the need for prevention and encourage individuals to take precautions [151].

**Seasonal campaigns:** Launch focused advertising during high-risk seasons, such as winter, when the usage of heating appliances raises CO exposure risks [152].

***Goals of public awareness campaigns:*** Public awareness campaigns seek to increase knowledge by educating people on CO poisoning sources, symptoms, and early detection methods; promote preventive action by encouraging regular maintenance of combustion appliances and the installation of CO detectors; and empower communities by providing individuals with the knowledge to recognise symptoms and seek timely medical intervention [153].

## 9. Future Perspectives

### 9.1. Advances in Diagnostics and Therapeutics

Continued research aims to increase the accuracy and efficiency of CO poisoning diagnostic tools, including the creation of new biomarkers and sophisticated COHb detection technologies. Therapeutic advances, such as optimising hyperbaric oxygen therapy regimens and investigating adjunct medicines, show promise for improving patient outcomes [154].

### 9.2. Development of Portable Detection Devices

The development of small, portable CO detectors with higher sensitivity and real-time monitoring capabilities will transform early detection. Wearable sensors and smartphone-integrated technology can help with point-of-care testing, ensuring rapid intervention in both resource-rich and resource-constrained environments [155].

### 9.3. Strategies for Enhanced Healthcare Delivery

Improving CO poisoning management requires strengthening healthcare facilities, particularly in poor places. This involves educating healthcare workers, extending emergency services, and introducing telemedicine for remote diagnosis and consultation. Furthermore, policy-driven actions to increase CO detector availability and public education can ensure broad preventative measures [156].

## 10. Conclusions

Carbon monoxide poisoning poses significant diagnostic and therapeutic challenges due to its non-specific symptoms and the variability in clinical presentation. While traditional methods such as carboxyhaemoglobin measurement and hyperbaric oxygen therapy are critical, their limitations highlight the need for advancements in portable diagnostic tools and personalised management approaches. Emerging technologies, including portable CO-oximeters and novel biomarkers, hold promises for enhancing early detection and prognosis. To improve outcomes, healthcare systems must prioritise accessibility to advanced diagnostic tools and timely treatment, especially in resource-limited settings. Future research should focus on refining protocols and integrating modern technologies into clinical practice.

## Figures and Tables

**Table 1:** Overview of Diagnostic Tests for Carbon Monoxide Poisoning: Principles, Performance, and Limitations.

Test Name	Principle	Development Time	Advantages	Sensitivity	Limitations	Ref.
CO-Oximetry (Spectrophotometry)	Differentiates COHb from oxyhaemoglobin using multiple wavelengths of light.	2–5 min	Accurate and direct measurement of COHb levels.	High	Requires specialised equipment; not widely available in all settings.	[58]
Blood Gas Analysis with CO-Oximetry	Measures COHb using arterial or venous blood and co-oximetry.	5–10 min	Simultaneous measurement of blood gases and COHb.	High	Time-sensitive; levels decrease quickly after oxygen therapy.	[121]
Non-Invasive Pulse CO-Oximetry	Uses multiwavelength light to non-invasively measure COHb.	Real-time	Non-invasive, rapid, and suitable for point-of-care testing.	Moderate to high	Less accurate at very low or very high COHb levels.	[122]
Standard Pulse Oximetry	Measures oxygen saturation using two wavelengths of light.	Real-time	Readily available and easy to use.	Low	Cannot distinguish between oxyhaemoglobin and carboxyhaemoglobin (false normal).	[123]
Breath CO Analysis	Measures exhaled CO levels as a proxy for COHb concentration.	1–2 min	Non-invasive, simple, and portable.	Moderate	Limited accuracy; influenced by recent exposure and respiration rate.	[124]

## Data Availability

No new data were created or analyzed in this study. Data sharing is not applicable to this article.
